# Priming of the cGAS-STING-TBK1 Pathway Enhances LPS-Induced Release of Type I Interferons

**DOI:** 10.3390/cells10040785

**Published:** 2021-04-01

**Authors:** Alessandra Tesser, Giulia Maria Piperno, Alessia Pin, Elisa Piscianz, Valentina Boz, Federica Benvenuti, Alberto Tommasini

**Affiliations:** 1 Department of Pediatrics, Institute for Maternal and Child Health—IRCCS Burlo Garofolo, 34137 Trieste, Italy; alessandra.tesser@burlo.trieste.it (A.T.); alessia.pin@burlo.trieste.it (A.P.); elisa.piscianz@burlo.trieste.it (E.P.); 2 International Centre for Genetic Engineering and Biotechnology (ICGEB), 34149 Trieste, Italy; Giulia.Piperno@icgeb.org (G.M.P.); Federica.Benvenuti@icgeb.org (F.B.); 3 Department of Medicine, Surgery, and Health Sciences, University of Trieste, 34149 Trieste, Italy; valentina.boz@burlo.trieste.it

**Keywords:** type I interferons, Toll-like receptor 4, DNase2-deficiency, interferonopathy, Systemic Lupus Erythematosus

## Abstract

Cytoplasmic nucleic acids sensing through cGAS-STING-TBK1 pathway is crucial for the production of antiviral interferons (IFNs). IFN production can also be induced by lipopolysaccharide (LPS) stimulation through Toll-like receptor 4 (TLR4) in appropriate conditions. Of note, both IFN production and dysregulated LPS-response could play a role in the pathogenesis of Systemic Lupus Erythematosus (SLE). Indeed, LPS can trigger SLE in lupus-prone mice and bacterial infections can induce disease flares in human SLE. However, the interactions between cGAS and TLR4 pathways to IFNs have been poorly investigated. To address this issue, we studied LPS-stimulation in cellular models with a primed cGAS-STING-TBK1 pathway. cGAS-stimulation was naturally sustained by undigested self-nucleic acids in fibroblasts from DNase2-deficiency interferonopathy, whilst it was pharmacologically obtained by cGAMP-stimulation in THP1 cells and murine bone marrow-derived dendritic cells. We showed that cells with a primed cGAS-STING-TBK1 pathway displayed enhanced IFNs production after TLR4-challenge. STING-inhibition did not affect IFN production after LPS alone, but prevented the amplified IFN production in cGAMP-primed cells, suggesting that functional STING is required for priming-dependent enhancement. Furthermore, we speculated that an increased *PIK3AP1* expression in DNase2-deficient fibroblasts may link cGAMP-priming with increased LPS-induced IFN production. We showed that both the hyper-expression of *PIK3API* and the enhanced LPS-induced IFN production can be contrasted by STING inhibitors. Our results may explain how bacterial LPS can synergize with cGAS-pathway in promoting the development of SLE-like autoimmunity.

## 1. Introduction

Triggering cytoplasmic sensors with nucleic acids results in inflammatory response dominated by type I interferon (IFN) production, considered as a physiological and conserved defense mechanism against viral infections [1].

Although several proteins can recognize nucleic acids in the cytoplasm, only the cyclic GMP-AMP synthase (cGAS) has emerged as the crucial sensor in all experimental settings [2], as well as in distinct mendelian IFN-related disorders [3]. cGAS senses cytoplasmic double-stranded DNA and DNA:RNA hybrids [4], catalyzing the production of the dinucleotide cyclic GMP-AMP (cGAMP) [5], a second messenger that activates the STING protein, which is considered the principal stimulator of IFN genes. From STING activation, the signal conveys on the TBK1 kinase (TANK-Binding Kinase 1), which induces the activation of the IFN Regulatory Factors IRF3/7 and the production of type I IFN [6,7].

TBK1-mediated production of type I IFN can also be triggered by the stimulation of Toll-like receptor 4 (TLR4) with bacterial lipopolysaccharide (LPS) in murine models of autoimmunity [8]. Mounting evidence supports a role for TLR stimulation by bacterial LPS in the pathogenesis of Systemic Lupus Erythematosus (SLE), both in mice and in humans. Indeed, it is well recognized that bacterial infections can trigger disease relapses in SLE [9,10,11].

Recent data showed that the priming of cGAS-STING-TBK1 pathway to IFNs can sensitize cells to an enhanced production of interleukin-1 upon stimulation with LPS, highlighting a complex crosstalk between distinct inflammatory pathways [12]. Furthermore, LPS can induce cGAS expression resulting in improved IFN response to cytosolic double-stranded nucleic acids [13]. We recently observed that fibroblasts with DNase2-deficiency display enhanced IFN production upon LPS stimulation. Starting from this model, we hypothesized a role of the priming of cGAS-STING-TBK1 pathway in the observed hyper-production of IFN upon LPS stimulation and developed cellular models to further analyze the crosstalk between the cGAS-STING-TBK1 and the TLR4 pathways in IFN inflammation. Our results highlight the role of the adapter protein BCAP, encoded by *PI3KAP1*, in connecting the two pathways. These findings can be relevant for a better understanding of the mechanisms underlying IFN overproduction after bacterial stimulus in lupus-models and the development of new complementary therapeutic strategies.

## 2. Materials and Methods

### 2.1. Fibroblasts

Fibroblasts of a patient with DNase2 interferonopathy (D2I) and from healthy control subject were maintained at a density of about 2 × 10^4^ cell/cm^2^ with complete medium (RPMI with 10% FBS, 100 U/mL Penicillin, 100 μg/mL Streptomycin, 2 mM L-Glutamine, all from EuroClone; Milan, Italy), supplemented with Normocin (100 μg/mL, InvivoGen, San Diego, CA, USA). For all experiments, fibroblasts were used at a density of 10^4^ cell/cm^2^ with complete medium (with 10% low endotoxins FBS, Microtech, Naples, Italy).

Fibroblasts were treated for 1 h with scalar concentrations of LPS (0.5–1–5 μg/mL, *E*. *coli*—serotype 055:B5, Sigma-Aldrich, St. Louis, MO, USA).

For phospho-TBK1 evaluation after STING inhibition, fibroblasts were pre-incubated for 2 h with human STING inhibitor H-151 (2.5–5–10 μM, InvivoGen), and afterward treated for 1 h with LPS (0.5 μg/mL, Sigma-Aldrich).

For IFN signature assessment and *PIK3AP1* expression (after 24, 48 and 72 h treatment with H-151 10 μM), fibroblasts were recovered for RNA extraction (High Pure miRNA Isolation Kit, Roche, Basel, Switzerland) and cDNA synthesis (SensiFAST™ cDNA Synthesis Kit, Bioline, London, UK), following the manufacturer’s instructions.

### 2.2. Interferon Signature and PIK3AP1 Quantification

IFN signature by Y. Crow is performed on whole blood and consist of six IFN-stimulated genes: *IFI27*, *IFI44L*, *IFIT1*, *ISG15*, *RSAD2, SIGLEC1*. For our experiments, we run a five-genes IFN signature, because *SIGLEC1* expression in fibroblasts is often too low to be detected. The IFN signature analysis and *PIK3AP1* expression were assessed by qPCR using AB 7500 Real Time PCR System (Applied Biosystems, Waltham, MA, USA), TaqMan Gene Expression Master Mix (Applied Biosystems) and UPL Probes (Roche). Using AB 7500 Real Time PCR software, each target quantity was normalized with the expression level of *HPRT1* and *G6PD*, and the relative quantification was conducted relating to the calibrator sample (control fibroblasts) using the 2^−∆∆Ct^ method [14].

### 2.3. Phospho-TBK1 Evaluation by Flow Cytometry

Fibroblasts were firstly fixed with PBS without Ca/Mg with 4% paraformaldehyde for 10 min and after permeabilized with 500 μL of cold 90% methanol. After washing, fibroblasts were stained for 1 h in the dark at room temperature with the anti-pTBK1 antibody (pTBK1/NAK1, ser172, clone D52C2, rabbit, PE, final concentration 4 μg/mL, Cell Signaling Technology, Danvers, MA, USA) or with the isotype control (rabbit DA1E mAb IgG XP™ isotype control, PE, final concentration 4 μg/mL, Cell Signaling Technology), and recovered to proceed with cytofluorimetric analysis on MacsQuant (Miltenyi Biotec, Bergisch Gladbach, Germany). Results were expressed as median fluorescence intensity (MFI) compared to isotype control after morphologic gating on live fibroblasts.

### 2.4. “IFN Priming” Model in THP1-Dual™ Reporter Cells

THP1-Dual™ cells (InvivoGen) are derived from the human THP1 monocyte cell line by stable integration of secreted inducible reporter construct to monitor type I IFN pathway activation. THP1-Dual™ cells were pre-incubated for 24 h with scalar concentrations of 2′3′-cGAMP (0.5–1.25–2.5–5–10–20 μg/mL, InvivoGen), and then challenged for 1 h with LPS (0.5 μg/mL, Sigma-Aldrich).

Type I IFN pathway activation was measured according to the manufacturer’s instructions.

### 2.5. Mouse Bone Marrow-Derived Dendritic Cells

Mouse bone marrow-derived dendritic cells (BMDCs) were generated in vitro from bone marrow of C57BL/6 WT mice using granulocyte macrophage colony-stimulating factor (GM-CSF). Dendritic cells were cultured at the concentration of 1.5 × 10^6^ cells/mL in not-treated 6 well-plates for 7 days using IMDM medium supplemented with 10% FBS, 50 μM 2-Mercaptoethanol, and 1% Gentamicin, complemented with 30% of supernatant of GM-CSF produced from J558 cell line. Cells were used for experiments on day 7.

For STING inhibition, BMDCs were pre-incubated for 1 h with 0.5 µM of murine STING inhibitor C-178 (ProbeChem, Shanghai, China) or the equivalent concentration of DMSO. After, stimuli were added to the wells without washing the inhibitor.

Cell culture supernatants were collected after 16 h for type I IFN quantification.

### 2.6. Type I IFN Quantification

B16-Blue IFN-α/β™ cells are specifically designed for type I IFN quantification (InvivoGen). The IFN production assays of BMDCs were performed according to the manufacturer’s instructions (InvivoGen). IFNβ (Biolegend, San Diego, CA, USA) (0–20 ng/mL) was added to generate a standard curve.

### 2.7. Transcriptomic Analysis

Transcriptome sequencing was performed using the TruSeq Stranded Total RNA with Ribo-Zero H/M/R Gold Sample Preparation kit (Illumina, San Diego, CA, USA) and sequenced on a NovaSeq 6000 platform (Illumina), generating 2 × 100 bp paired-end reads (60 million reads per sample) in D2I fibroblasts (3 replicates) and in control fibroblasts (2 replicates).

RNAsequencing (RNAseq) raw data workflow was conducted as follows: quality control by FastQC (https://www.bioinformatics.babraham.ac.uk/projects/fastqc/, fastqc 08-01-19: v0.11.9 released), quality filtering by Trimmomatic (v.0.39) [15] read alignment to GRChg38 (primary assembly) using annotation from GENECODE v.35 (GTF, comprehensive gene annotation, PRI) (https://www.gencodegenes.org/) with STAR (v.2.7.6a) [16], reads counting into genes by featureCounts (v.2.0.1) [17]. Most variable genes across samples were selecting running rowVars function in R. Data were normalized and analyzed for differentially expressed genes by DESeq2 (v.1.30.1) [18]. The results table was generated according to the default parameters except for alpha = 0.05. Representative genes have been selected by fold change greater than 2-fold increase/decrease and adjusted *p*-value < 0.05, according to the Benjamini–Hochberg method [19].

### 2.8. Pathway Analysis

Selected differentially expressed genes have been analyzed for pathway enrichment running the enrichKEGG function by the R package clusterProfiler (v.3.18.1) [20] with the default parameters and according to the KEGG database for over-representation test analysis.

Visualization of functional enrichment results was performed with cnetplot function by the clusterProfiler package and by R package enrichplot (v.1.10.2).

### 2.9. Statistics

Data analysis was performed using GraphPad Prism 8 software with Two-way ANOVA; results were reported as mean ± SEM of independent experiments; *p*-values < 0.05 were considered statistically significant.

### 2.10. Study Approval

The study is part of the IRCCS Burlo Garofolo project RC#24/2017, approved by the Institutional Review Board and by the Friuli Venezia Giulia Independent Ethical Committee (2018-SPER-079-BURLO, N. 0039851, approved on 12 December 2018). Written informed consent was received from the participant prior to inclusion in the study.

## 3. Results

### 3.1. DNase2-Deficient Fibroblasts Show a Constitutive IFN Pathway Activation, and a Greater IFN-Response after LPS Stimulation Than Controls

**Figure 1:**
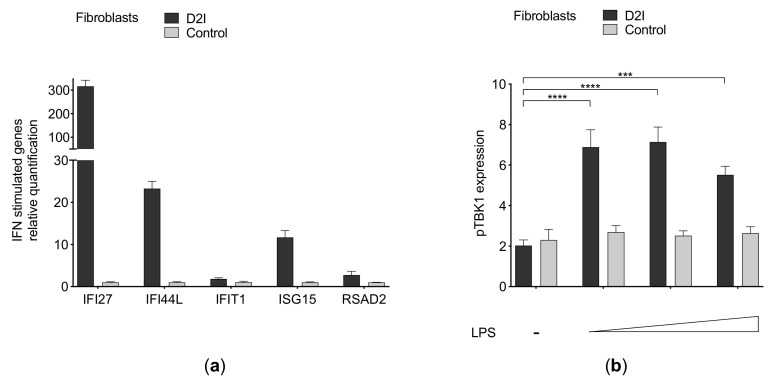
(**a**) Interferon (IFN) signature in DNase2-deficient (D2I) and control fibroblasts. Relative quantification by qPCR of IFN-stimulated genes (*IFI27, IFI44L, IFIT1, ISG15, RSAD2*) normalized on housekeeping genes (*G6PD, HPRT1*). (**b**) Flow cytometry assessment of phospho-TBK1 in D2I and control fibroblasts after LPS challenge (0.5–1–5 μg/mL) for 1 h (*n* = 3 independent experiments). Results are reported as mean ± SEM of median fluorescence intensity of pTBK1 expression compared to isotype control. Statistically significant *p*-values are shown (Two-way ANOVA: *** *p* < 0.001; **** *p* < 0.0001).

Fibroblasts from DNase2 interferonopathy (D2I) present a constitutive primed IFN signaling, witnessed by increased IFN signature compared with the control (Figure 1a). In D2I fibroblasts, LPS stimulation led to dramatically increased activation of IFN signaling, as measured by TBK1 phosphorylation (Figure 1b).

### 3.2. Priming of the cGAS-STING-TBK1 Pathway Is Associated with Increased Activation of Type I IFN Pathway after LPS Challenge in a Human Monocytic Cell Line and in Murine Bone Marrow-Derived Dendritic Cells

We hypothesized that the effect observed in D2I fibroblasts could be due to a permissive effect of an activated cGAS pathway on the TLR4-TBK1 pathway. Consistently, we investigated the effect that cGAS-pathway priming can exert on LPS-induced production of IFN in other distinct cell models: a human monocyte-derived cell line engineered with a report system that allows to track IFN activation by a luminescent reaction (THP1-Dual™ reporter cells), and murine bone marrow-derived dendritic cells (BMDCs).

To stimulate the cGAS-STING-TBK1 pathway in a similar manner as in D2I, we used the second mediator 2′3′ cyclic guanosine monophosphate–adenosine monophosphate (cGAMP), which directly activates STING, mimicking the effect of cGAS stimulation.

**Figure 2:**
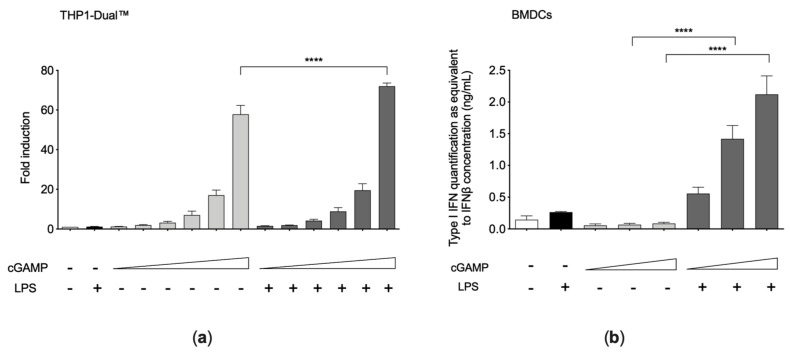
(**a**) Type I interferon (IFN) pathway activation in THP1-Dual™ cells after cGAMP and LPS challenges (*n* = 4 independent experiments). THP1-Dual™ cells were pre-treated for 24 h with cGAMP (0.5–1.25–2.5–5–10–20 μg/mL), and afterward stimulated for 1 h with LPS (0.5 μg/mL). Luminescence values were normalized on the untreated condition. (**b**) Type I IFN production in bone marrow-derived dendritic cells (BMDCs) after cGAMP and LPS challenges (*n* = 3 independent experiments). BMDCs were pre-treated for 24 h with cGAMP (0.025–0.5–1 μg/mL) and afterward stimulated for 16 h with LPS (1 μg/mL). Secreted type I IFN was evaluated by B16-Blue IFN-α/β™ reporter cells assay. Results are reported as mean ± SEM. Statistically significant *p*-values are shown (Two-way ANOVA: **** *p* < 0.0001).

24 h cGAMP priming in THP1-Dual™ cells led to an increase of LPS-induced type I IFN production compared with cGAMP priming alone or LPS stimulation alone, as assessed both by the internal reporter (Figure 2a) and by measure of IFN in supernatants with HEK-Blue™ IFN-α/β reporter cells. This effect was barely detectable in THP1-Dual™, but much more evident in BMDCs (Figure 2b), which are known for their strong capability of activating the IFN pathway.

### 3.3. STING Inhibition Prevents the IFN Hyper-Activation after LPS Challenge in cGAMP-Primed BMDCs and in D2I Fibroblasts

**Figure 3:**
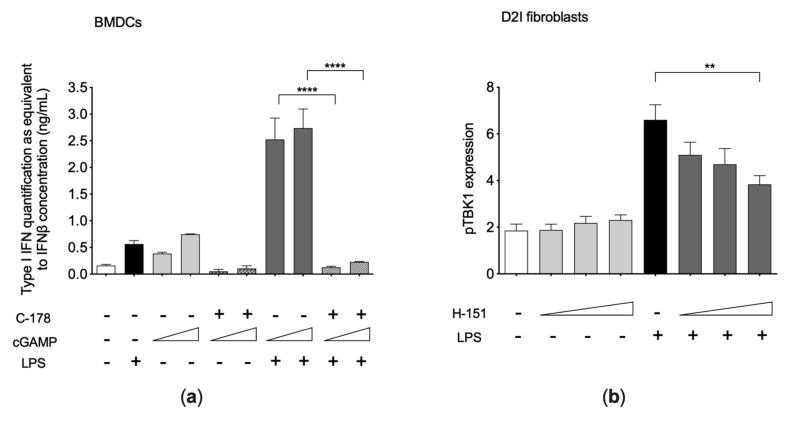
(**a**) Type I interferon (IFN) production in bone marrow-derived dendritic cells (BMDCs) after cGAMP, C-178, and LPS challenges (*n* = 4 independent experiments). BMDCs were treated as follows: incubated for 24 h with cGAMP (0.025–1 μg/mL); washed and treated for 1 h with C-178 (0.25 μM); and stimulated for 16 h with LPS (1 μg/mL). Secreted type I IFN was evaluated by B16-Blue IFN-α/β™ reporter cells assay. (**b**) Phospho-TBK1 assessment in D2I fibroblasts after H-151 treatment and LPS stimulation (*n* = 3 independent experiments). D2I fibroblasts were pretreated for 2 h with human STING inhibitor H-151 (2.5–5–10 μM) and afterward stimulated for 1 h with LPS (0.5 μg/mL), without washing the inhibitor. Results are reported as mean ± SEM of median fluorescence intensity of pTBK1 expression compared to isotype control. Statistically significant *p*-values are shown (Two-way ANOVA: ** *p* < 0.01; **** *p* < 0.0001).

We showed that a treatment with the murine STING inhibitor (C-178) strongly reduced the LPS-induced IFN release from cGAMP pre-treated BMDCs (Figure 3a), confirming the crucial role of functional STING in the cGAMP-primed LPS response. Likewise, a treatment with the human STING inhibitor H-151 considerably reduced LPS-induced phosphorylation of TBK1 (*p*-value = 0.0055) in D2I fibroblasts (Figure 3b).

### 3.4. Transcriptomics Analysis of D2I Fibroblasts

We performed a transcriptomic study in D2I fibroblasts to assess if the observed susceptibility to LPS-induced IFN production might be associated with altered expression of proteins involved in the pathways leading from cGAS and TLR4 stimulation to the IFN production. No significant change was noted in the components of the cGAS-STING-TBK1 pathway (Table 1). Conversely, we could detect a ~24 fold increased expression of the *PIK3AP1* gene, which is involved in the modulation of the TLR4 pathway to IFN (see below).

**Figure 4:**
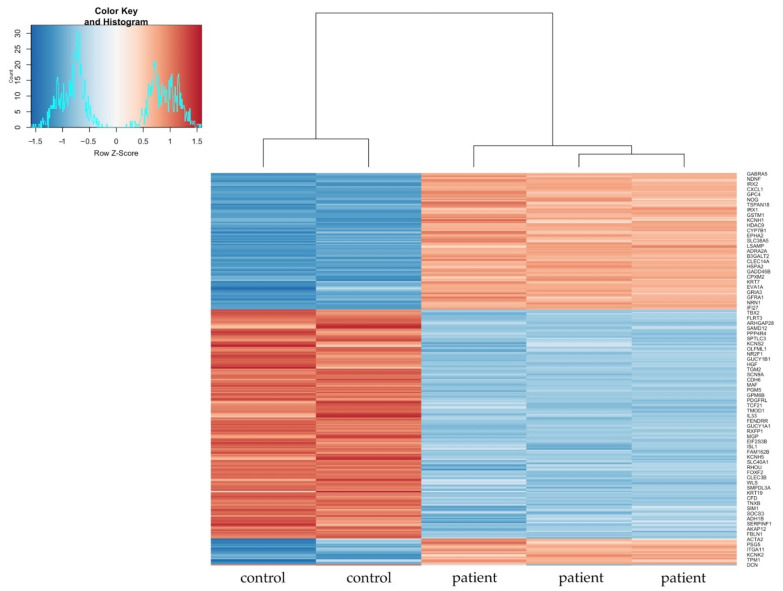
Heatmap of the most variable genes across all samples. Hierarchical clustering shows a distinct gene expression pattern of DNase2-deficient fibroblasts (D2I, “patient”, *n* = 3 replicates) compared to control (“control”, *n* = 2 replicates). In red positive values of z-score and in blue negative values of z-score.

Figure 4 displays the heatmap of the most variable genes across all samples. The hierarchical clustering shows a distinct gene expression pattern of D2I fibroblasts compared to control.

**Figure 5:**
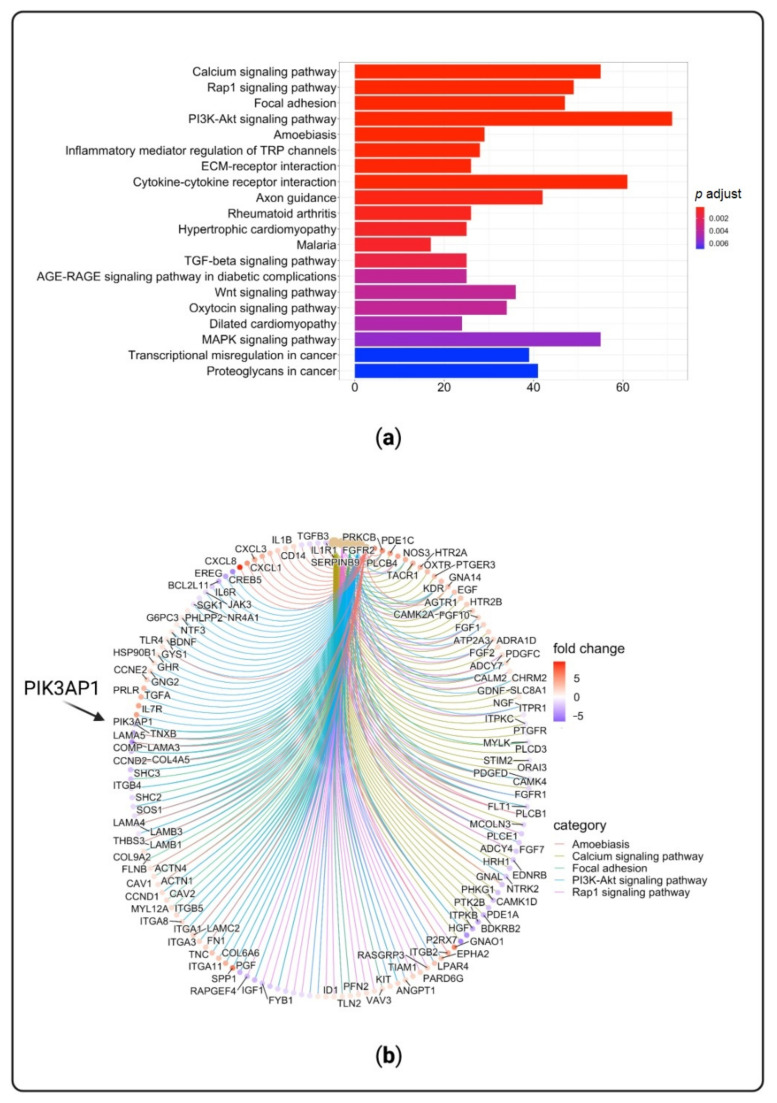
Over-represented pathways and networks of significantly differentially expressed genes (DEGs) in DNase2-deficient fibroblasts (D2I). (**a**) Twenty most enriched pathways that are ordered by *p*-values displayed by gradient color scale that represents the full range of *p*-values (lowest *p*-values in red). (**b**) Functional enrichment results of up-regulated and down-regulated genes between D2I and control fibroblasts. Fold change refers to log2FoldChange values (in red up-regulated genes and in violet down-regulated genes).

Pathway analyses were run on significantly differentially expressed genes (DEGs) between D2I and control fibroblasts. Figure 5a reports the 20 most significant over-represented functional pathways of significantly DEGs. Figure 5b shows the up-regulated and down-regulated genes involved in the five most enriched pathway among which is PI3K-AKT signaling pathway, which includes *PIK3AP1* gene.

*PIK3AP1* encodes for an adaptor protein named BCAP, which is involved in the modulation of TLR-mediated type I IFN induction [21], in the tuning of PI3K/AKT axis signaling [22,23,24], and in the modulation of macrophage differentiation. The potential influence of BCAP protein in the development of IFN-producing macrophages and autoimmune B cells suggests a possible role in SLE pathogenesis [21].

This evidence suggests that the *PIK3AP1* gene could be a logical candidate to explain the hyper-activation of the IFN pathway observed in response to LPS in our models.

### 3.5. STING Inhibition Prevents PIK3AP1 Hyper-Expression 

**Figure 6:**
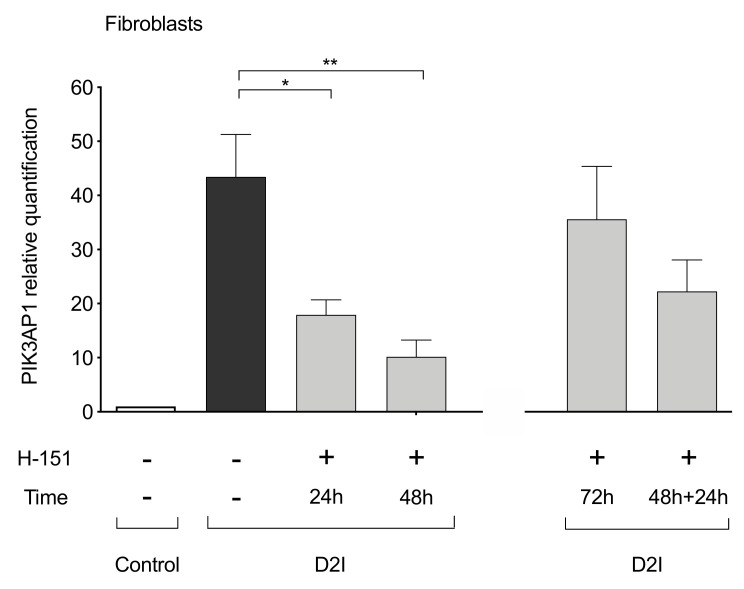
Relative quantification by qPCR of *PIK3AP1* in DNase2-deficient (D2I) and control fibroblasts (*n* = 5 independent experiments). D2I fibroblasts were incubated for 24 h and 48 h with human STING inhibitor H-151 (10 μM) and afterward collected for RNA extraction and *PI3KAP1* quantification. On the right, results after 72 h of H151 (10 μM) incubation, and 48 h plus 24 h H151 (10 μM) re-inoculation. Results are reported as mean ± SEM. Statistically significant *p*-values are shown (Two-way ANOVA: * *p* < 0.05; ** *p* < 0.01).

The qPCR analysis confirmed an hyper-expression of *PIK3AP1* gene in D2I fibroblasts, which could be greatly reduced by a treatment of 24 h (*p*-value = 0.0280) and 48 h (*p*-value = 0.0026) with human STING inhibitor (H-151) (Figure 6). After 72 h stimulation with H-151, *PIK3AP1* expression gets back closer to untreated values, probably due to H-151 deterioration in medium. Indeed, *PIK3AP1* expression is reduced after the re-stimulation with H-151 for 24 h following the initial 48 h of treatment. Taken together, these experiments confirmed that intact STING is necessary for *PIK3AP1* expression.

We thus hypothesize that *PIK3AP1* hyper-expression may link the cGAMP-priming with the amplification of LPS-induced secretion of IFNs.

## 4. Discussion

Our study builds on the serendipitous observation of an hyper-activation of IFN release in D2I fibroblasts stimulated with LPS. D2I is a monogenic SLE-like disorder due to the defective disposal of nucleic acids in lysosomes, which results in stimulation of the cGAS-STING-TBK1 pathway and IFN-mediated inflammation [25]. Starting from this observation, we hypothesize that the priming of the cGAS-STING-TBK1 pathway may sensitize the cells to the production of IFN upon LPS stimulation. Indeed, the enhanced LPS-induced IFN release in D2I fibroblasts requires intact STING functioning, as it could be blocked by STING inhibition. Although our in vitro study was conducted on the cGAS-STING-TBK1 pathway priming performed before LPS challenge, we cannot determine in vivo the consequentiality of signaling activations, which may be due to a “synergic” or a “sequential” effect. Further studies may be needed to clarify this aspect.

We showed that priming of the cGAS-STING-TBK1 pathway has a permissive effect on LPS-induced IFN release also in other cellular models, specifically a human monocyte-derived cell line (THP1-Dual™ reporter) and murine BMDCs. Monocytes and dendritic cells are key players in the pathogenesis of systemic autoimmune diseases and SLE [26]. In the presence of IFNs, monocytes differentiate to dendritic cells that potentiate and sustain autoreactive lymphocytes [27]. Similarly, BMDCs can be defined somewhat as IFN production practitioners, given their recognized role in the production of IFNs and in driving the production of autoantibodies [28]. We showed that priming with cGAMP, which mimics the effect of undigested double strand cytoplasmic nucleic acids seen in D2I, led to enhanced IFN response to LPS in a similar manner. Further experiments in BMDCs demonstrated that STING is required for priming but not for response to LPS alone. This is in agreement with previous data linking TLR4 stimulation with an activation pathway converging on TBK1, apparently not requiring STING. However, our data suggest that, albeit STING is not required to mediate LPS-induced IFN in normal conditions, it is indispensable for cGAMP-primed enhancement of IFN production upon stimulation with LPS.

These data may be relevant to the pathogenesis of SLE, as defective nuclease activity or an increased release of nucleic acids from the cell (e.g., due to mitochondrial or nuclear damage) or from phagocytized apoptotic cells have been associated with IFN-driven inflammation [29,30,31]. Accordingly, abnormal exposition to self-DNA can enhance LPS-induced polyclonal B cell proliferation and autoantibody production [31]. Several murine models support a role for bacterial LPS in promoting and enhancing autoantibody production [32,33,34] and, in humans, it has been shown that increased plasma levels of LPS correlate with the production of anti-double-stranded DNA antibodies in relatives of patients affected with SLE [21].

We also tried to uncover the molecular basis of cGAS-STING-TBK1 priming effect in D2I fibroblasts or in cells stimulated with cGAMP. First, we showed that the priming effect in D2I fibroblasts is not associated with constitutive hyper-phosphorylation of TBK1. Then, we analyzed the transcriptomic profile of D2I fibroblasts to search for possible changes related to the hyper-responsivity to LPS. By focusing on the two pathways involved in cGAMP and LPS signaling, the only gene with significantly altered expression in D2I fibroblast was *PIK3AP1*, coding BCAP, an adaptor protein coupling various receptors, like B cell receptor and Toll-like receptors, to PI3K [35]. BCAP is a major modulator of TLR signaling, downregulating NF-kB activation [36] and favoring type I IFN induction [21]. Very recent data showed that activation of the STING-Lyn pathway leads to *PIK3AP1* hyper-expression, which can be relevant to the development of SLE in lupus prone mice, thus adding substance to our findings [37]. The BCAP impact on the transition of macrophages from a “proinflammatory state” to a “reparative state” could influence the development of IFN-producing macrophages and autoimmune B cells [38]. BCAP protein has also been associated with the tuning of PI3K signaling [23] and with the regulation of PI3K/AKT pathway activation by TLRs stimulation [24], affecting whether the stimulation results in activation of the NF-kB or the IFN pathway. The fine tuning of PI3K activation has a crucial role in the immune system, impacting maturation, proliferation, and survival of immune cells [39].

Our results confirmed that STING is necessary for *PIK3AP1* hyper-expression in D2I cells, as *PIK3AP1* expression could be substantially reduced by STING-inhibitor. Thus, we can hypothesize that STING-dependent hyper-expression of *PIK3AP1* may make cGAMP primed cells prone to LPS-induced release of IFNs. Of course, this is only a candidate biological correlate of the observed priming effect, and we cannot exclude that other epigenetic factors (methylation, phosphorylation, ubiquitination) of signal proteins have a more important role. Infact, we have yet to definitively prove that *PIK3AP1* is needed to address the TLR signal toward the production of IFNs, because the precise mechanisms linking *PIK3AP1* expression to the modulation of TLR-signaling are not completely elucidated.

## 5. Conclusions

Undigested nucleic acids and bacterial stimulation may cooperate in a positive loop resulting in dysregulated IFN production. The understanding of the mechanisms underlying IFN over-production after bacterial stimulus in lupus-models may be relevant for the development of new complementary therapeutic strategies. Overall, our results provide a possible functional link between the role of nucleic acids and LPS in the pathogenesis of SLE.

## Figures and Tables

**Table 1:** Expression of genes involved in the cGAS-STING-TBK1 axis.

Gene	Log2FoldChange	*p* Adjust ^A^
*STING*	−0.17	0.59
*CGAS*	−0.03	0.95
*TBK1*	0.11	0.74
*PIK3AP1*	4.63	1.48 × 10^−7^

^A^ Adjusted *p*-value according to the Benjamini-Hochberg method [19].

## Data Availability

Raw data will be provided upon reasonable request.

## References

[1] Stetson D.B., Medzhitov R. (2006). Type I interferons in host defense. Immunity.

[2] Vance R.E. (2016). Cytosolic DNA Sensing: The Field Narrows. Immunity.

[3] Gao D., Li T., Li X.D., Chen X., Li Q.Z., Wight-Carter M., Chen Z.J. (2015). Activation of cyclic GMP-AMP synthase by self-DNA causes autoimmune diseases. Proc. Natl. Acad. Sci. USA.

[4] Wu J., Sun L., Chen X., Du F., Shi H., Chen C., Chen Z.J. (2013). Cyclic GMP-AMP is an endogenous second messenger in innate immune signaling by cytosolic DNA. Science.

[5] Sun L., Wu J., Du F., Chen X., Chen Z.J. (2013). Cyclic GMP-AMP synthase is a cytosolic DNA sensor that activates the type I interferon pathway. Science.

[6] Honda K., Yanai H., Negishi H., Asagiri M., Sato M., Mizutani T., Shimada N., Ohba Y., Takaoka A., Yoshida N. (2005). IRF-7 is the master regulator of type-I interferon-dependent immune responses. Nature.

[7] Schafer S.L., Lin R., Moore P.A., Hiscott J., Pitha P.M. (1998). Regulation of type I interferon gene expression by interferon regulatory factor-3. J. Biol. Chem..

[8] Sakaguchi S., Negishi H., Asagiri M., Nakajima C., Mizutani T., Takaoka A., Honda K., Taniguchi T. (2003). Essential role of IRF-3 in lipopolysaccharide-induced interferon-beta gene expression and endotoxin shock. Biochem. Biophys. Res. Commun..

[9] Manfredo Vieira S., Hiltensperger M., Kumar V., Zegarra-Ruiz D., Dehner C., Khan N., Costa F.R.C., Tiniakou E., Greiling T., Ruff W. (2018). Translocation of a gut pathobiont drives autoimmunity in mice and humans. Science.

[10] Ma Y., Xu X., Li M., Cai J., Wei Q., Niu H. (2019). Gut microbiota promote the inflammatory response in the pathogenesis of systemic lupus erythematosus. Mol. Med..

[11] Azzouz D., Omarbekova A., Heguy A., Schwudke D., Gisch N., Rovin B.H., Caricchio R., Buyon J.P., Alekseyenko A.V., Silverman G.J. (2019). Lupus nephritis is linked to disease-activity associated expansions and immunity to a gut commensal. Ann. Rheum. Dis..

[12] Swanson K.V., Junkins R.D., Kurkjian C.J., Holley-Guthrie E., Pendse A.A., El Morabiti R., Petrucelli A., Barber G.N., Benedict C.A., Ting J.P. (2017). A noncanonical function of cGAMP in inflammasome priming and activation. J. Exp. Med..

[13] Ma F., Li B., Liu S.Y., Iyer S.S., Yu Y., Wu A., Cheng G. (2015). Positive feedback regulation of type I IFN production by the IFN-inducible DNA sensor cGAS. J. Immunol..

[14] Pin A., Monasta L., Taddio A., Piscianz E., Tommasini A., Tesser A. (2019). An Easy and Reliable Strategy for Making Type I Interferon Signature Analysis Comparable among Research Centers. Diagnostics.

[15] Bolger A.M., Lohse M., Usadel B. (2014). Trimmomatic: A flexible trimmer for Illumina sequence data. Bioinformatics.

[16] Dobin A., Davis C.A., Schlesinger F., Drenkow J., Zaleski C., Jha S., Batut P., Chaisson M., Gingeras T.R. (2013). STAR: Ultrafast universal RNA-seq aligner. Bioinformatics.

[17] Liao Y., Smyth G.K., Shi W. (2014). featureCounts: An efficient general purpose program for assigning sequence reads to genomic features. Bioinformatics.

[18] Love M.I., Huber W., Anders S. (2014). Moderated estimation of fold change and dispersion for RNA-seq data with DESeq2. Genome Biol..

[19] Benjamini Y., Hochberg Y. (1995). Controlling the False Discovery Rate: A Practical and Powerful Approach to Multiple Testing. J. R. Stat. Soc. Ser. B.

[20] Yu G., Wang L.G., Han Y., He Q.Y. (2012). clusterProfiler: An R package for comparing biological themes among gene clusters. OMICS.

[21] Chu T., Ni M., Chen C., Akilesh S., Hamerman J.A. (2019). Cutting Edge: BCAP Promotes Lupus-like Disease and TLR-Mediated Type I IFN Induction in Plasmacytoid Dendritic Cells. J. Immunol..

[22] Ruse M., Knaus U.G. (2006). New players in TLR-mediated innate immunity: PI3K and small Rho GTPases. Immunol. Res..

[23] Inabe K., Kurosaki T. (2002). Tyrosine phosphorylation of B-cell adaptor for phosphoinositide 3-kinase is required for Akt activation in response to CD19 engagement. Blood.

[24] Troutman T.D., Hu W., Fulenchek S., Yamazaki T., Kurosaki T., Bazan J.F., Pasare C. (2012). Role for B-cell adapter for PI3K (BCAP) as a signaling adapter linking Toll-like receptors (TLRs) to serine/threonine kinases PI3K/Akt. Proc. Natl. Acad. Sci. USA.

[25] Rodero M.P., Tesser A., Bartok E., Rice G.I., Della Mina E., Depp M., Beitz B., Bondet V., Cagnard N., Duffy D. (2017). Type I interferon-mediated autoinflammation due to DNase II deficiency. Nat. Commun..

[26] Wang J., Dai M., Cui Y., Hou G., Deng J., Gao X., Liao Z., Liu Y., Meng Y., Wu L. (2018). Association of Abnormal Elevations in IFIT3 With Overactive Cyclic GMP-AMP Synthase/Stimulator of Interferon Genes Signaling in Human Systemic Lupus Erythematosus Monocytes. Arthritis Rheumatol..

[27] Gkirtzimanaki K., Kabrani E., Nikoleri D., Polyzos A., Blanas A., Sidiropoulos P., Makrigiannakis A., Bertsias G., Boumpas D.T., Verginis P. (2018). IFNα Impairs Autophagic Degradation of mtDNA Promoting Autoreactivity of SLE Monocytes in a STING-Dependent Fashion. Cell Rep..

[28] Siegemund S., Hartl A., von Buttlar H., Dautel F., Raue R., Freudenberg M.A., Fejer G., Büttner M., Köhler G., Kirschning C.J. (2009). Conventional bone marrow-derived dendritic cells contribute to toll-like receptor-independent production of alpha/beta interferon in response to inactivated parapoxvirus ovis. J. Virol..

[29] Bodaño A., González A., Ferreiros-Vidal I., Balada E., Ordi J., Carreira P., Gómez-Reino J.J., Conde C. (2006). Association of a non-synonymous single-nucleotide polymorphism of DNASEI with SLE susceptibility. Rheumatology.

[30] Lu Q., Wang J.Y., Wang L., Jiang X., Chu Y. (2014). Self DNA from lymphocytes that have undergone activation-induced cell death enhances murine B cell proliferation and antibody production. PLoS ONE.

[31] Richez C., Yasuda K., Watkins A.A., Akira S., Lafyatis R., van Seventer J.M., Rifkin I.R. (2009). TLR4 ligands induce IFN-alpha production by mouse conventional dendritic cells and human monocytes after IFN-beta priming. J. Immunol..

[32] Lee T.P., Huang J.C., Liu C.J., Chen H.J., Chen Y.H., Tsai Y.T., Yang W., Sun K.H. (2014). Interactions of surface-expressed TLR-4 and endosomal TLR-9 accelerate lupus progression in anti-dsDNA antibody transgenic mice. Exp. Biol. Med..

[33] Lee T.P., Tang S.J., Wu M.F., Song Y.C., Yu C.L., Sun K.H. (2010). Transgenic overexpression of anti-double-stranded DNA autoantibody and activation of Toll-like receptor 4 in mice induce severe systemic lupus erythematosus syndromes. J. Autoimmun..

[34] Ogunrinde E., Zhou Z., Luo Z., Alekseyenko A., Li Q.Z., Macedo D., Kamen D.L., Oates J.C., Gilkeson G.S., Jiang W. (2019). A Link Between Plasma Microbial Translocation, Microbiome, and Autoantibody Development in First-Degree Relatives of Systemic Lupus Erythematosus Patients. Arthritis Rheumatol..

[35] Yamazaki T., Kurosaki T. (2003). Contribution of BCAP to maintenance of mature B cells through c-Rel. Nat. Immunol..

[36] Lauenstein J.U., Scherm M.J., Udgata A., Moncrieffe M.C., Fisher D.I., Gay N.J. (2020). Negative Regulation of TLR Signaling by BCAP Requires Dimerization of Its DBB Domain. J. Immunol..

[37] Thim-Uam A., Prabakaran T., Tansakul M., Makjaroen J., Wongkongkathep P., Chantaravisoot N., Saethang T., Leelahavanichkul A., Benjachat T., Paludan S.R. (2020). STING Mediates Lupus via the Activation of Conventional Dendritic Cell Maturation and Plasmacytoid Dendritic Cell Differentiation. iScience.

[38] Irizarry-Caro R.A., McDaniel M.M., Overcast G.R., Jain V.G., Troutman T.D., Pasare C. (2020). TLR signaling adapter BCAP regulates inflammatory to reparatory macrophage transition by promoting histone lactylation. Proc. Natl. Acad. Sci. USA.

[39] Katso R., Okkenhaug K., Ahmadi K., White S., Timms J., Waterfield M.D. (2001). Cellular function of phosphoinositide 3-kinases: Implications for development, immunity, homeostasis, and cancer. Annu. Rev. Cell Dev. Biol..

